# Role of Copper and Cholesterol Association in the Neurodegenerative Process

**DOI:** 10.1155/2013/414817

**Published:** 2013-10-29

**Authors:** Nathalie Arnal, Gustavo R. Morel, María J. T. de Alaniz, Omar Castillo, Carlos A. Marra

**Affiliations:** ^1^INIBIOLP (Instituto de Investigaciones Bioquímicas de La Plata), CCT La Plata, CONICET-UNLP, Cátedra de Bioquímica y Biología Molecular, Facultad de Ciencias Médicas, Universidad Nacional de La Plata, 60 y 120 (1900) La Plata, Argentina; ^2^CIC (Centro de Investigaciones Cardiovasculares), CCT La Plata, CONICET-UNLP, Facultad de Ciencias Médicas, Universidad Nacional de La Plata, 60 y 120 (1900) La Plata, Argentina

## Abstract

Age is one of the main factors involved in the development of neurological illnesses, in particular, Alzheimer, and it is widely held that the rapid aging of the world population is accompanied by a rise in the prevalence and incidence of Alzheimer disease. However, evidence from recent decades indicates that Cu and Cho overload are emerging causative factors in neurodegeneration, a hypothesis that has been partially investigated in experimental models. The link between these two variables and the onset of Alzheimer disease has opened up interesting new possibilities requiring more in-depth analysis. The aim of the present study was therefore to investigate the effect of the association of Cu + Cho (CuCho) as a possible synergistic factor in the development of an Alzheimer-like pathology in Wistar rats. We measured total- and nonceruloplasmin-bound Cu and Cho (free and sterified) contents in plasma and brain zones (cortex and hippocampus), markers of oxidative stress damage, inflammation, and programmed cell death (caspase-3 and calpain isoforms). The ratio beta-amyloid (1-42)/(1-40) was determined in plasma and brain as neurodegenerative biomarker. An evaluation of visuospatial memory (Barnes maze test) was also performed. The results demonstrate the establishment of a prooxidative and proinflammatory environment after CuCho treatment, hallmarked by increased TBARS, protein carbonyls, and nitrite plus nitrate levels in plasma and brain zones (cortex and hippocampus) with a consequent increase in the activity of calpains and no significant changes in caspase-3. A simultaneous increase in the plasma A**β**1-42/A**β**1-40 ratio was found. Furthermore, a slight but noticeable change in visuospatial memory was observed in rats treated with CuCho. We conclude that our model could reflect an initial stage of neurodegeneration in which Cu and Cho interact with one another to exacerbate neurological damage.

## 1. Introduction

The aging of the world population is being accompanied by a rise in the prevalence and incidence of Alzheimer disease (AD) and other neurodegenerative illnesses. In the USA, the number of patients with AD is expected to increase to 13 million by 2050 and in the European Union (EU) to over 4 million [1]. The mortality rate of AD is second only to cancer and stroke. Epidemiologic data indicates that the world population will have grown considerably by 2025 and the percentage of elderly people will be significantly higher [2]. Since age is one of the major risk factors in the development of AD, it is expected that the incidence of this disease will also increase. However, experimental evidence detailed in a review by Brewer indicates that other factors in addition to age could play a critical role [3] in the development of diseases such as Alzheimer and that the contribution of inorganic copper (Cu) has been underestimated. It is widely known that more than 95% of AD cases are sporadic and only 2–7% are genetically determined [4]. Thus, any environmental factors likely to have an etiopathogenic role in AD should be investigated.

Though Cu is essential to human health, Cu overload has been associated with mental decline [5] and particularly with AD development [3, 6, 7]. Data from Squitti's group specifically demonstrated that free Cu (also known as NCBC or nonceruloplasmin-bound Cu) is elevated in the blood of AD patients, negatively correlates with cognition, and predicts the rate of loss of cognition [8–10]. More recently, our group corroborated these findings in an independent human cohort and demonstrated that increased NCBC has a direct impact on the disease duration [11]. We also proposed the NCBC/ceruloplasmin ratio as a predictive marker of risk for the first-degree relatives of AD patients. Sparks and Schreurs [12] first demonstrated that Cu supplementation in drinking water given to rabbits under a diet with excess cholesterol (Cho) produced an induction of *β*-amyloid plaques and a learning deficit. In addition, Lu et al. reported that trace amounts of Cu activate the apoptotic cascade and exacerbate beta amyloid-induced neurotoxicity in Cho-fed mice through a TNF-mediated inflammatory pathway [13, 14]. Very recently, Brewer has reviewed the theory of inorganic Cu toxicity in Alzheimer disease as a causative factor in cognitive loss [6]. However, the question of how inorganic Cu might trigger a neurodegenerative process is still a matter of debate [15].

The degree of exposure of human populations to Cu is also a controversial issue. There is little data on Cu overload in humans since most of the available evidence (experimental or epidemiological) was obtained from animal models. However, the regulatory framework for chronic Cu exposure in large human populations indicates that pollution, drinking water, and dietary Cu-containing supplements are the main sources of exposure [6, 16]. The dietary reference intake for people in the USA, United Kingdom, Europe, and Australia varies from 0.16 to 0.98 (Estimated Average Requirements) EAR or (Recommended Dietary Allowance) RDA expressed in mg Cu/kg body weight, with considerable variations as a function of age. The (Population Reference Intake) PRI was reported between 0.3 and 1.5 mg Cu/kg body weight [16]; however, these limits were largely surpassed in many circumstances such as ingestion of fish, bivalves, or contaminated drinking water [3, 17]. In accumulated data on 280 samples of household drinking water all across North America, 72% of the samples have Cu levels above those enhancing AD in experimental models [3]. The content of inorganic Cu in dietary supplements can be as high as 3 mg/pill (approx. 2 mg above the EAR or RDA). Furthermore, we and others have demonstrated significantly higher Cu levels in plasma of women using Cu-IUDs and in blood samples from farmers working with Cu-based pesticides [17–19]. Most importantly, we found alterations in Cu homeostatic biomarkers in neurodegenerative patients and their first-degrees relatives [11].

Fat ingestion has also been linked to AD prevalence [20]. Specifically, Cho has been associated with oxidative stress and AD development [21, 22], with most of the experimental evidence emerging from exploration of the role of Cho in *β*-amyloid formation [23]. Despite the paucity of epidemiological data from human studies, it is reasonable to assume that Cho plays at least some role in learning and memory and is associated with AD pathogenesis [24]. Of particular interest is evidence showing that Cu greatly exacerbates cognitive decline in those people included in the highest quintile of fat ingestion [25]. Experimental evidence obtained from rabbit and mouse models suggests that the association of Cu and Cho can be risk factor for AD development [12–14]. Moreover, it was hypothesized that Cu could oxidize Cho, generating substances toxic to the brain [3].

However, the mechanism of action of Cu and Cho in AD incidence and development is poorly understood and requires further investigation. Thus, the aim of this work was to study the effects of Cu and Cho association on the two main brain areas affected in AD, cortex and hippocampus, using a model of Wistar rats. Specifically, we aimed to determine for each nutritional supplement alone or in combination (i) the capacity to install oxidative/nitrative damage; (ii) changes in the levels of the main antioxidant molecules (glutathione and *α*-tocopherol); (iii) the possible development of a pro-inflammatory condition by analyzing the concentration of prostaglandins PGE2 and PGF2*α*; (iv) the activities of the two main protease systems associated with programmed cell death, caspase-3 and calpains (*μ*- and m-); and (v) possible changes in visuospatial memory as assessed by means of the Barnes maze test. 

Our findings could be useful in further investigating the mechanisms underlying the neurodegenerative process and also in localizing putative targets for preventive interventions associated with endogenous and/or exogenous causative factors such as Cu and Cho, alone or in combination.

## 2. Material and Methods

### 2.1. Chemicals

All chemicals used were of analytical grade and obtained from Sigma Chem. Co. (Buenos Aires, Argentina or USA), Merck (Darmstadt, Germany), and Carlo Erba (Milan, Italy).

### 2.2. Animals and Treatments

Certified pathogen-free male Wistar rats were used. The rats were maintained at a controlled temperature (25°C) and relative humidity of 60% with forced ventilation, under a normal photoperiod of 12 h darkness and 12 h light. The health of the animals was monitored in accordance with the internationally recommended practices of the (*Institute of Laboratory Animal Resources, Commission of Life Sciences, National Research Council*) ILAR. Solid food and drinking water were provided *ad libitum*. The diets for the experiments were prepared in our laboratory according to the recommendations for Wistar rats [26]. All procedures for handling the animals followed the NIH regulations [27]. The experimental protocol was reviewed and approved by (Bioethics Committee of the Faculty of Medical Sciences, UNLP) COBIMED under the code # 00382/11.

### 2.3. Experimental Protocols

Rats (21 days old) were randomly assigned (ten animals per group) to the protocols detailed as follows and treated during eight weeks. The control group (C) was maintained on lab-prepared pellets as recommended for normal growth, containing 7 ppm of Cu [28, 29]. The Cu-supplemented experimental group (Cu) was fed on control pellets and tap water supplemented with 3 mg/L (or ppm) of Cu in the form of ultrapure CuSO_4_ (Merck, Darmstadt, Germany), the Cho-supplemented group (Cho) was fed on pellets containing 2% (W/W) of Cho (87% pure) (obtained from Saporiti SRL, Buenos Aires, Argentina), and the Cu + Cho-supplemented group (CuCho) was simultaneously treated with Cu in water + Cho in food. Rats were monitored during the experimental period to observe their behavior, quantify water, and food consumption and determine their body weight gain. Total Cu concentration in tap water supplemented with CuSO_4_ was determined by means of atomic absorption methodology and was 3.42 ± 0.21 ppm (means of all daily measurements along the experimental period). Considering that each animal imbibed between 4.9 ± 0.4 and 15.0 ± 1.1 mL water/day (at the beginning and the end of the protocol, resp.), a maximum of 0.01 to 0.05 mg Cu/day was acquired from water (a dose equivalent to 0.06 and 0.18 mg Cu/Kg live animal, resp.). Linear regression curves and ANOVA test for Cu content in food demonstrated that there were no significant variations between the 6 preparations used for the experiments (7.22 ± 0.31 ppm or mg Cu/Kg diet). Fe and Zn content (determined by atomic absorption spectrometry) were the same in all preparations (45.9 ± 1.8 and 66.6 ± 2.0 ppm, resp.). Ingestion of solid food along the experiments varied from 11.6 ± 0.8 to 29.7 ± 2.8 g/rat, implying that the oral ingestion of Cu was in the range of 0.08 to 0.21 mg Cu/day/rat (0.90 to 1.21 mg Cu/Kg live animal, a mean of 1.06 ± 0.11 mg Cu/Kg).

### 2.4. Sample Collection

At the end of the treatments, animals were deeply anesthetized with ketamine (70 mg/Kg) and xylazine (5 mg/Kg) applied intramuscularly and then sacrificed by decapitation. Brains were rapidly taken out and dissected in two zones, cortex and hippocampus, using the atlas of Paxinos and Watson [30] as a guide for tissue dissection and a Binocular Stereoscopic Arcano Ztx-1065 Microscope (Instrumental Pasteur, Buenos Aires, Argentina). Both brain regions were washed, weighed, and homogenized using a buffer Tris/HCl (10 mM pH 7.4) with sucrose (70 mM), mannitol (230 mM), ethylenediaminetetraacetic acid (EDTA) (1 mM), and dithiothreitol (DTT) (1 mM). 

### 2.5. Atomic Absorption Measurements

Aliquots of sample were digested with a mixture of 4 mL of HNO_3_ (c) and 1 mL HClO_4_ (Aldrich or Sigma Chem. Co., Buenos Aires, Argentina) by heating at 120°C for 80 min in a mineralization block [31]. The digests were cooled, diluted with ultrapure water (18 mΩ cm, Carlo Erba, Milan, Italy), and ultrafiltered by a 0.22 *μ*m Millipore membrane (Milli-Q Purification System, from Millipore, CA, USA). Ultrafiltered dissolutions were directly aspirated into the flame of a Perkin-Elmer 1100 B Spectrophotometer equipped with a Perkin-Elmer cathode lamp (Perkin-Elmer Corp., Norwalk, CT, USA) at a spectral width of 1 nm. Standard solutions of 100 ppm from HCR Inc. (QuimiNet, Buenos Aires, Argentina) were used for Zn and Fe. Cu determinations were calibrated with a standard solution (200 ppm) of Cu (NO_3_)_2_ in HNO_3_ 0.5 N (Tritrisol from Merck Co., Darmstadt, Germany). All measurements were carried out in peak height mode (324.7 nm line). The intra-[(SD/x)·100] and inter-[(ΔSD/Δx)·100] assay coefficients of variation were 15.5 and 6.0%, respectively. We routinely obtained a similar equation for the calibration curve (IR = 55.10^−5^ + 0.048·[Cu, mg/L]), and the statistical analyses demonstrated a correlation coefficient always between 0.95 and 0.99. In addition, we explored the so-called matrix effects that could have modified the slopes of the standard regressions. In spiked samples the obtained values varying from 48 to 60.10^−5^ were very similar to those of Cu standard solutions, indicating that the matrix effect was not significant or was negligible. The mean for recovery and RSD for spiked samples was 99.7% and 3.3%, respectively, and the detection limit was 0.09 mg/L. In order to verify the accuracy of the method, we explored the influence of time after dilution, temperature of acid digestion, and concentration of HNO_3_/HClO_4_ following the suggestions of Terrés-Martos et al. [32]. We also checked our results with biological samples (plasma and homogenates) against a Seronorm Trace Elements Serum (from Sero Labs, Billingstad, Norway) and found no significant differences between the obtained and the declared (certified) concentrations.

### 2.6. Ceruloplasmin (CRP) Levels and Nonceruloplasmin-Bound Copper (NCBC)

Samples were analyzed by the enzyme conversion of p-phenylenediamine into a blue-colored product [33] which was then measured at 550 nm. Reaction proceeded at 37°C in buffer glacial acetic/sodium acetate (50 mM, pH 5.5) directly into flat-bottomed plates, using a Microplate Reader SpectraMax M2/m2^e^ model from Molecular Devices Analytical Technologies (Sunnyvale, CA, USA) for 3 min. Intra- and interassay coefficients of variation were 8.3 and 4.4%, respectively. CRP concentrations were calculated by comparison with the reaction rate of pure CRP standard (Sigma Chem. Co., Buenos Aires, Argentina). Using the Cu and CRP data, we calculated the non-CRP-bound Cu (NCBC, or so-called free Cu) as described by Brewer [3] by subtracting the amount of Cu bound to each mg of CRP from data of total Cu. This parameter can be easily expressed in percentages using the formula (([Cu]-47.2 × [CRP]) × 100/[Cu]), where Cu is in *μ*mol/L and CRP is in g/L [34].

### 2.7. Lipid Analysis

Total lipids were extracted by the method of Folch et al. [35]. Aliquots of this solution were taken to measure total Cho by an enzymatic method using a commercial kit from Wienner Lab (Rosario, Argentina). To estimate the amount of esterified Cho (ECho), aliquots of the lipid extracts were seeded in high-resolution pre-coated silica gel plates (10 × 20 cm) with a concentration zone for thin layer chromatography (Whatman Adsorption 60 Å Silica Gel HP-TLC Plates, CA, USA) and developed with diethyl ether : hexane : acetic acid (90 : 4 : 1, by vol) as described elsewhere [36]. Authentic standards of Cho and Cho-esthers (from Avanti Polar Lipids, Ontario, Canada) were run in parallel and revealed by iodine vapors. Identified zones were scraped off the plates, eluted with Folch reactive, evaporated, dissolved in 50 mM phosphate buffer (pH 7.4) with 1% sodium deoxycholate, and enzymatically analyzed using the commercial kits from Wienner Lab (Rosario, Argentina).

### 2.8. Biomarkers of ROS Production

Thiobarbituric acid-reactive substances (TBARS) were measured in brain homogenates as previously described [37]. TBARS (mainly malondialdehyde, MDA) reacted with 2-thiobarbituric acid (TBA) to yield TBA-MDA adducts which were quantified at 532 nm. The concentration of the chromophore was calculated from a calibration curve prepared with fresh tetramethoxypropane (TMP) solutions (TMP was purchased from Sigma Chem. Co., Buenos Aires, Argentina). Nitrate and nitrite [NOx] concentration were measured using the method of Griess on samples previously reduced with vanadium chlorohydrate according to Miranda et al. [38]. Quantification was performed after calibration with standard solutions of sodium nitrate from Merck Co. (Darmstadt, Germany). Protein carbonyls (PCs) were determined by the method of Reznick and Packer [39]. The concentrations of PCs were calculated from a calibration curve prepared with a stock solution of sodium pyruvate (Sigma Chem. Co.).

### 2.9. Antioxidant Defense System

#### 2.9.1. Reduced (GSH) and Oxidized (GSSG) Glutathione Content

Total glutathione was determined by the glutathione reductase/dithionitrobenzoic (DTNB) method that can measure both GSH and GSSG [40]. To calculate the GSH/GSSG ratio, samples were reanalyzed after derivatization with divinylpyridine (3 mM final concentration).

#### 2.9.2. Vitamin E (*α*-tocopherol)

Vitamin E (*α*-tocopherol) was measured after extraction with the Buttriss and Diplock method [41] using the HPLC technique of Bagnati et al. [42] in a reverse phase/C-18 silica column from ALLTECH Associates, Inc. (Deerfield, IL, USA). The ECONOSIL C_18_ column with a Direct-connect Cartridge Guard Column System was operated at a maximum pressure of 500 psi in a Hitachi HPLC System (Tokyo, Japan). The amount of vitamin was electronically calculated using internal calibration with pure *α*-tocopherol (Sigma, Bs. As.), and the results were expressed in *μ*M concentration of *α*-tocopherol.

#### 2.9.3. Glutathione Reductase (GR)

GR activity was determined by the method of Carlberg and Mannervik [43]. The specific activity of the enzyme was calculated for each sample in terms of U/min·mg protein (*ε* = 6.22 nM^−1^·cm^−1^ for absorbance at 340 nm).

### 2.10. Biomarkers of Inflammation

 Prostaglandin F2*α* (PG F2*α*) and prostaglandin E2 (PGE2) were measured using the PGF2*α* EIA Kit and PGE2 Express EIA Kit, respectively (Cayman, Migliore Laclaustra S.R). The results were expressed as ng of each prostaglandin/mg total protein.

### 2.11. Programmed Cell Death Biomarkers

#### 2.11.1. Caspase-3 Activity

Caspase-3 activity was measured by a colorimetric assay kit (CASP-3-C) based on the hydrolysis of the synthetic peptide substrate acetyl-Asp-Glu-Val-Asp-p-nitroaniline (Ac-DEVD-pNA) by caspase-3 (Sigma Chem. Co., Buenos Aires, Argentina). The resulting p-nitroaniline (p-NA) was monitored at 405 nm. Each assay was run in parallel with inhibitor-treated homogenates (to measure the nonspecific hydrolysis of the substrate) and caspase-3 positive control (using commercial caspase-3, 5 mg/mL provided by the kit manufacturer). A calibration curve using a standard solution of p-nitroaniline (p-NA) was also run for each assay to calculate the activity of the protease expressed as *μ*mol p-NA released/min·mg protein.

#### 2.11.2. Milli- (m-) and Micro- (*μ*-) Calpains

The assay involves the hydrolysis of whole ultrapure casein (Sigma, Chem. Co., CA, USA) by calpain activity and the subsequent detection of trichloroacetic acid (TCA) soluble peptidic fragments at 280 nm [44]. To select the activity of the calpain isoforms, the level of calcium in the medium was regulated (5 mM or 500 *μ*M of CaCl_2_ for m- or *μ*-calpain, resp.). The activity of calpains was calculated considering that a unit of calpain is the amount of enzyme that produces a change of absorbance of 0.01 at 280 nm. Results were expressed as units/min·mg of protein. 

### 2.12. Markers of Neurodegeneration

#### 2.12.1. Beta Amyloid Peptides (1-40) and (1-42)

Beta amyloid peptides (A*β*) (1-40) and (1-42) were measured using Human/Rat *β* Amyloid-40 ELISA kit Wako II and the Human Amyloid-42 ELISA kit Wako High-Sensitivity, respectively. The A*β*(1-42)/(1-40) ratio was then calculated from the individual data expressed as picomole of the respective A*β* peptide/mg total protein.

#### 2.12.2. Barnes Maze Test

The Barnes maze is a black acrylic circular platform, 122 cm in diameter and elevated 108 cm off the floor, containing twenty holes around the periphery. The 10 cm diameter holes are uniform in appearance but, one hole is connected to an escape box, consisting of a 38.7 cm long × 12.1 cm wide × 14.2 depth cm removable box. Four proximal visual cues (30 cm high, opaque black geometric figures: a cross, a circle, a square, and a triangle) were located in the room 50 cm from the circular platform. The escape box remained in a fixed position relative to the cues, to ensure randomization of the hole associated with the escape tunnel. In the center of the platform is a starting chamber (an opaque, 26 cm diameter, 20 cm high, and white plastic open-ended cylinder). A 90 dB white-noise generator and a white-light 500 W bulb provided motivation for escaping from the platform. The acquisition session and the probe trial session were performed on the same day. In brief, the experiment consisted of eight acquisition trials (t1–t8) followed by a single evaluation trial (t9). Acquisition trials began with the animal inside the starting chamber for 30 seconds in the presence of a constant buzz. The chamber was then raised, the aversive stimulus (intense bright light) was switched on, and the rat was allowed to freely explore the maze. The rats were each given 120 s to locate the correct hole. If by the end of this period they had not entered the escape box of their own accord, they were gently picked up and placed over the hole above the escape box. The evaluation trial proceeded in the same manner as described above but without the start box. At the end of each trial, the aversive stimulus was switched off, the rat remained on the escape box during 60 s, and the white light was switched off. In order to eliminate any possible olfactory clues from the maze, it was cleaned with 10% ethyl alcohol solution at the beginning of the 15 min intertrial period. An individual hole exploration was defined as being a single downward head deflection toward the inside of the hole. The following parameters were determined: (i) first-hole latency time (in s) spent by the animal between being released from the start box and exploring a hole in the maze for the first time; (ii) escape-box latency time (defined in the acquisition and retention test trials as the time (in s) spent by the animal between being released from the start box and entering the escape box and, in the case of the preference test and extinction trials, the time elapsed before the first exploration of the escape hole); and (iii) nongoal hole exploration (defined as the number of explorations of holes other than the escape hole, the explorations being considered as errors during the acquisition and probe trials). In the case of the evaluation trials, we evaluated the hole exploration frequency (the number of explorations of each hole during the trial in which the escape hole was numbered as hole 0 for normalized graphical representation purposes, 1 to 10 clockwise, and −1 to −9 counterclockwise). The behavioral measurements were recorded using a video camera mounted 110 cm above the platform, linked to a computer. The video performances of each rat were analyzed using the video analysis software Kinovea-Creative Commons Attribution (v 0.7.6).

### 2.13. Statistical Analysis

All values represent the mean of 6 rats assayed in triplicate expressed as mean ± standard deviation (SD). Data were analyzed by ANOVA plus Tukey test with the aid of SPSS 11.0.1 software (SPSS Inc., Chicago, IL). To analyze the data from the Barnes maze test, multiple comparisons were drawn with the control group using two-way ANOVA plus the Holm-Sidak *post hoc* test at two levels of significance (*P* < 0.05 and 0.01). Data were also analyzed using MANOVA with identical final conclusions. Results were also plotted and analyzed using Sigma Scientific Graphing Software (version 11.0) from Sigma Chem. Co. (St. Louis, MO). The statistical significance of differences is indicated by distinct superscript letters (data with distinct superscript letters are statistically different (*P* < 0.01) between them). 

## 3. Results

### 3.1. Cholesterol and Copper Levels

Table 1 shows the levels of total and free Cu (NCBC or nonceruloplasmin-bound Cu) in plasma and in brain cortex and hippocampus after the experimental treatments. Plasma total Cu and NCBC exhibited discrete (but statistically significant) increases in those groups receiving Cu (Cu and CuCho). Total Cu also increased in both cortex and hippocampus after Cu supplementation (alone or in combination with Cho). Cortex shows a nonsignificant tendency towards higher NCBC after Cu or CuCho treatments, whereas hippocampus shows a significant increase in this parameter with respect to the control group. 

The results of the Cho analysis (free and esterified) are shown in Table 2, from which it is evident that tissue from the central nervous system has a particular Cho metabolism characterized by higher levels of free Cho (25 times higher than in the case of plasma). Samples from brain and plasma behave differently from one another. Cu addition to drinking water produced no significant changes in plasma total Cho and a slight increase in cortex and hippocampus at the expense of the esterified form (ECho). Furthermore, Cu did not change the ratio between free and ECho in plasma samples but did lower this parameter in the brain homogenates. Supplementing food with Cho caused a higher level of total Cho in peripheral plasma (from 27.0 ± 0.5 in the control group to 33.7 ± 0.6 nmoles/mg protein in Cho-supplemented rats) with a slight but significant decrease in the proportion of free/Echo. In brain, all the treatments led to an increase in total (free + esterified) Cho with respect to control data, though the increase was substantially higher and differentially orchestrated in those groups receiving Cho supplementation. In the case of Cho-treated rats, this occurred at the expense of free Cho; however, in rats under CuCho treatment, it was at the expense of the ECho form. Under CuCho treatment, hippocampus reached even higher values of total Cho than cortex (approx. 311 versus 295 nmoles/mg protein). Addition of Cu alone significantly elevated the ECho in both brain tissues, more noticeably in hippocampus than in cortex. Another differential result for the CuCho group was a decrease in free Cho with respect to control rats in cortex, a phenomenon not observed in hippocampus. The results obtained for the ratios between free and ECho strongly suggest that Cu addition was able to modify the balance of these two parameters, triggering the accumulation of ECho in cortex and hippocampus, perhaps facilitating its esterification or impeding its degradation, or both. This effect is especially noticeable under conditions of simultaneous Cu and Cho overload and, even more interestingly, was contrary to the effect observed in peripheral plasma samples.

### 3.2. Biomarkers of Oxidative Stress

**Figure 1:**
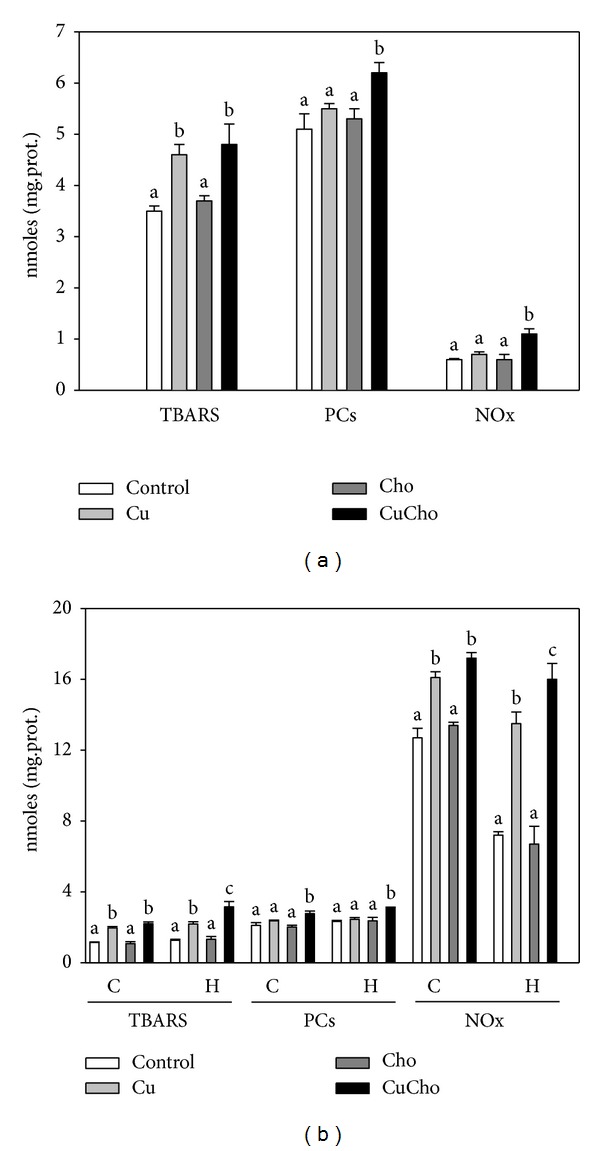
Oxidative/nitrative stress biomarkers in plasma (a) and brain cortex and hippocampus (b). Lipid peroxidation (TBARS) (nmol MDA/mg protein), protein oxidation (PCs) (nmol MDA/mg protein), and concentration of nitrites and nitrates ([NOx]) (*μ*mol/mg protein) were determined in plasma and brain cortex and hippocampus following the procedures described in described in Material and Methods Section. Treatments are indicated with different colors. Control (white bars), Cu (gray bars), Cho (dark gray bars), and CuCho (black bars). Results are expressed as mean of 10 rats assayed in triplicate ± standard deviation (SD). Significant differences were indicated by letters (data with distinct letters are statistically different between them at *P* < 0.01).

In order to detect whether inorganic Cu in drinking water and Cho in food produced oxidative/nitrative stress, we measured the oxidation end products of lipids (TBARS) and proteins (PCs) and also the levels of NOx (nitrates and nitrites derived from the spontaneous dismutation of nitric oxide) as biomarkers of damage. Levels of the three markers analyzed were higher after CuCho treatment both in plasma and in brain cortex and hippocampus (Figures 1(a) and 1(b)). In plasma, treatment with Cu alone increased TBARS levels (Figure 1(a)), whereas all three biomarkers were significantly increased after CuCho cosupplementation. Brain homogenates exhibited similar results, with increased levels of all three biomarkers after Cu or CuCho supplementation (with the solo exception of PCs in both brain regions, which increased only with simultaneous exposure to Cu and Cho).

We also measured the levels of the two main antioxidant molecules for water- and lipid-soluble cellular compartments (total glutathione-GSH+GSSG- and *α*-tocopherol, resp.) (Table 3). Plasma levels of total glutathione were higher in rats fed on Cu plus Cho supplements. In the same experimental group (CuCho), the *α*-tocopherol concentration was 27% lower than in the control data. In brain cortex, we also observed an increase in total glutathione after CuCho treatments and a simultaneous increment in the GSSG/GSH ratio as a consequence of a higher level of GSSG. Concomitantly, the activity of glutathione reductase (GR) in cortex was enhanced by Cu supplementation and even more so by simultaneous treatment with Cu + Cho. The level of *α*-tocopherol (*α*-Toc) decreased significantly only after CuCho treatment (approx. 17% compared to control data). In hippocampus, the behavior was very similar, with the exception of the increment in total glutathion content. Thus, hippocampus homogenates showed a significant increase in the GSSG/GSH ratio, activation of GR in Cu- and CuCho-treated rats, and a ca. 34% decrease in *α*-Toc content compared to control data, with this latter being observed only in the CuCho experimental group.

### 3.3. Markers of Inflammation

**Figure 2:**
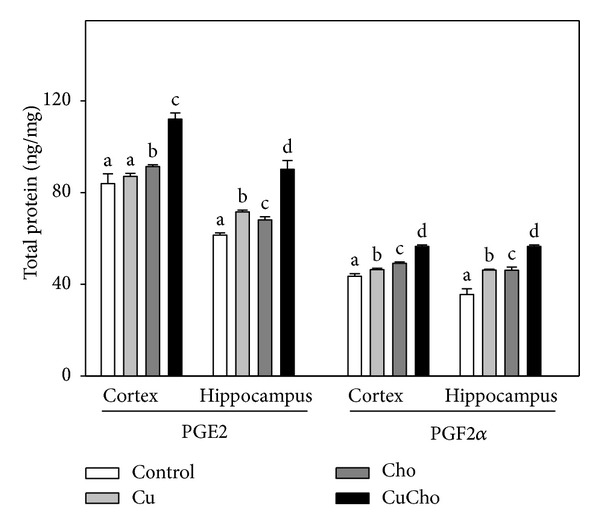
Prostaglandin PGF2*α* and PGE2 levels in brain cortex and hippocampus homogenates (ng/mg total protein). Treatments are indicated with different colors. Samples were analyzed as indicated in Section 2.10. Control (white bars), Cu (gray bars), Cho (dark gray bars), and CuCho (black bars). Results are expressed as mean of 10 rats assayed in triplicate ± standard deviation (SD). Significant differences were indicated by letters (data with distinct letters are statistically different between them at *P* < 0.01).

In order to evaluate whether simultaneous supplementation with Cu and Cho also produced an inflammatory condition, we analyzed the levels of two proinflammatory prostaglandins, PGE2 and PGF2*α* (Figure 2). In both brain regions, cosupplementation significantly increased prostaglandin levels. Interestingly, Cu and Cho alone also increased prostaglandins levels with respect to control data, and association of the two supplements produced an additive effect.

### 3.4. Caspase-3 and Calpains Activities

**Figure 3:**
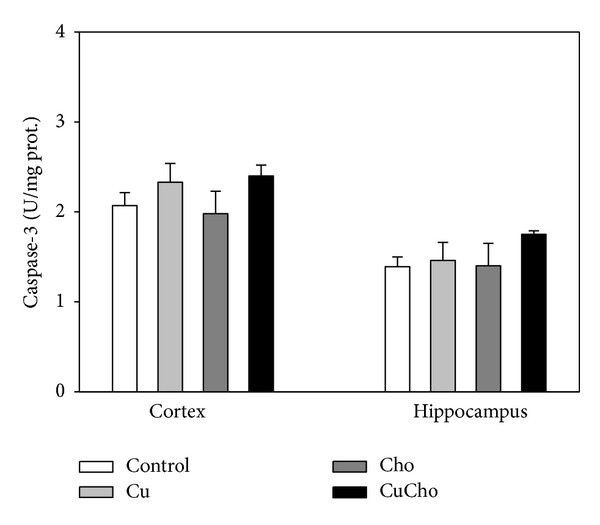
Caspase-3 (U/mg total protein) activity in brain cortex and hippocampus homogenates. Samples were analyzed according to the procedure described in Section 2.11.1. Treatments are indicated with different colors. Control (white bars), Cu (gray bars), Cho (dark gray bars), and CuCho (black bars). Results are expressed as mean of 10 rats assayed in triplicate ± standard deviation (SD). There are no statistically significant differences (*P* < 0.01) between treatments.

**Figure 4:**
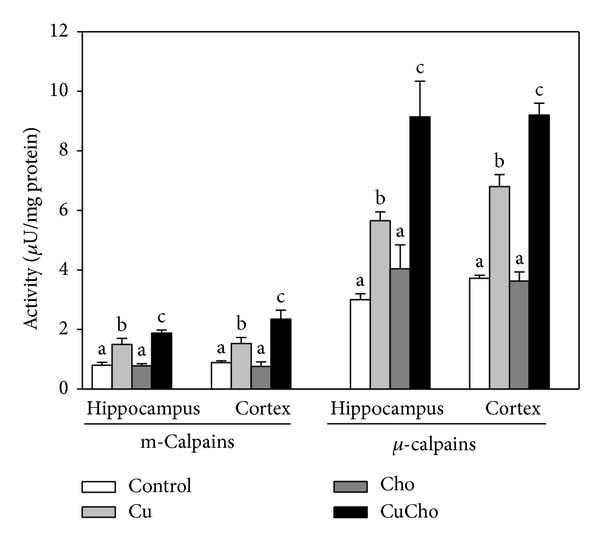
Calpains (*μ*- and m-) (U/min·mg total protein) activities in brain cortex and hippocampus homogenates. Samples were analyzed according to the procedures described in Section 2.11.2. Treatments are indicated with different colors. Control (white bars), Cu (gray bars), Cho (dark gray bars), and CuCho (black bars). Results are expressed as mean of 6 rats assayed in triplicate ± standard deviation (SD). Significant differences were indicated by letters (data with distinct letters are statistically different between them at *P* < 0.01).

We also explored whether the prooxidative and proinflammatory environment developed after CuCho treatment was able to trigger apoptotic signals, to which end we determined the activities of the two main protease systems involved in programmed cell death, caspase-3, and calpains (*μ*- and m-). Caspase-3 activity tends to increase at least in cortex homogenates, but not to a statistically significant degree (Figure 3). Both calpain (milli- and microisoforms) activities increased after Cu treatment and even more so after CuCho supplementation in both brain zones (Figure 4).

### 3.5. Biomarkers of Neurodegeneration

The concentration of the *β*-amyloid peptides (1-42 and 1-40) and the A*β* 1-42/1-40 ratio in cortex and hippocampus are shown in Table 4. The ratio (which is the main indicator of neurodegenerative process) was different, depending on the brain zone examined. In cortex, it was found to increase after Cu and Cho treatments and to further increase after CuCho supplementation. In hippocampus, the A*β* 42/40 ratio increased only in the CuCho experimental group. There were no statistically significant changes of the ratio (1-42)/(1-40) in peripheral plasma.

We also investigated possible alterations in the visuospatial learning capabilities through the Barnes maze test. We observed minor (not statistically different) changes in latency to the first hole and more spatial preference for the escape region (holes −1, 0, and 1) regardless of treatment (data not shown). Taken together, these changes demonstrate minor alterations in visuospatial memory suggesting that simultaneous supplementation with Cu and Cho produces an increment in exploratory activity—or a sort of overexcited behavior—but with similar final results to those observed in the other experimental groups. 

## 4. Discussion

Supplementation of drinking water with low amounts of inorganic Cu such as those used in our experiments was able to modify the basal status of redox biomarkers not only in peripheral plasma but also in the two zones of the central nervous system explored, cortex and hippocampus. Because of their short life span, free radicals cannot be measured directly, for which reason it is usual to analyze the products arising from reactions caused by reactive species with biomolecules. In line with this, we measured the end-oxidation products of lipids (TBARS) and proteins (PCs) and the production of NOx as biomarkers of prooxidative tissue damage. PCs increased only after CuCho treatment; however, TBARS and NOx increased after a sole exposure to trace amounts of inorganic Cu in drinking water. The biomarkers assayed were reproducible and sensitive to the experimental stimuli. Many authors have associated neurodegenerative illnesses with both a local (brain) and a systemic (plasma) increase in oxidative stress [45–52]. Irrespective of whether biomarkers are causative factors or whether they merely constitute phenomenologically associated changes, they are useful tools for evaluating the extent of damage and provide a simple methodology for monitoring large populations subjected to environmental Cu pollution or exposed to other risks associated with the development of AD. There is abundant evidence that transition metals in general, and specially Cu, are causative factors for oxidative stress and, as we mentioned previously, are strongly associated with the neurodegenerative process [53–56]. 

The decrease of *α*-tocopherol in plasma and brain zones homogenates and the increased GSSG/GSH ratio are both markers of accumulation of reactive species in lipid- and water-soluble cell compartments. The activation of GR can be interpreted as a compensatory mechanism of the enzymatic antioxidant defense system in order to normalize the altered GSSG/GSH ratio induced by cotreatment with Cu and Cho. In agreement with our results, Kojima et al. [57] have demonstrated the induction of mRNA coding for GR in the brain of mice irradiated with a low dose of *γ*-rays. Also in line with this, we can speculate that the observed increase in plasma total glutathione levels may be due to a compensatory mechanism under the oxidative insult evoked by the treatment studied here. This explanation is in agreement with the suggestions of other authors [58] and may be the consequence of an induction (overexpression) of cysteinyl-synthetase, the enzyme that controls the biosynthesis of glutathione [59, 60].

The oxidative stress induced by NCBC could have multiple effects on the signals cascade that depends on the redox state and can also modify the activity of ionic channels, transporters, and enzymes. Pallottini et al. [61] observed, in liver of Wistar rats treated with thioacetamide, that HMGCoA-reductase overactivation was strictly related to the magnitude of the reactive species accumulated. Our results demonstrate that inorganic Cu supplementation—even at the low levels assayed—produced an increase in ECho levels in cortex and hippocampus. Attributing the increase in Cho in brain to oxidative stress-induced HMGCoA-reductase hyperactivity could be oversimplistic, and this intriguing question remains to be resolved on the basis of new experimental evidence. Cho in the nervous system (10-fold higher than in any other organ) is mainly unesterified [62, 63]. Furthermore, most of the Cho content in brain depends on the *in situ* biosynthesis that appears to be regulated by similar mechanisms both outside and inside the brain, with HMGCoA-reductase being the most important regulatory effector [63]. However, the exact extent of Cho biosynthesis in neurons and astrocytes *in vivo* remains unknown [62], making it difficult to estimate the real effect of the accumulation of reactive species (NCBC-induced) on brain Cho biosynthesis. Interestingly, Cho turnover in individual neurons and astrocytes may in fact be very high and reach an estimated 20% per day, depending on the brain zone [62]. Unfortunately, the direct effect of higher Cho levels in blood on Cho concentration in brain is difficult to assess. Cho trafficking between brain and peripheral blood implies the participation of the blood-brain barrier (BBB) that hinders the direct passage into or out of the central nervous system. However, for reasons not yet understood, this restriction appears to be reduced in AD and other neurodegenerative disorders [64]. Also, in certain circumstances, for example, in cases of vascular injury due to oxidative stress, the BBB could be permeable to interaction with the peripheral pool of Cho [64]. Moreover, recent experimental evidence in a mouse model of AD demonstrated that inflammation is one of the key factors in determining increased BBB vulnerability [65]. Thus, from the above, we can assume that peripheral Cho might cross the BBB into the brain, in particular when the tissue is subjected to oxidative stress and redox-induced inflammation as observed in our experimental model. 

Despite extensive research in recent years, the role of Cho as a risk factor for AD remains controversial [64], likely due to the still unresolved questions relating to the exact role of peripheral Cho in its level in brain. We can speculate that NCBC induces nitrative/oxidative and pro-inflammatory conditions that probably facilitate endothelial damage and indirectly modify BBB properties [64, 65], allowing peripheral Cho to enter the central nervous system. Obviously, a great deal of experimental work is still required to either confirm or refute this working hypothesis. Several unresolved issues raise doubts concerning the beneficial effects of statins in neurodegenerative patients and the notion that high blood Cho is associated with brain dysfunction. However, one possibility is that the real risk is the association of hypercholesterolemia with a prooxidative and pro-inflammatory environment induced, for example, by NCBC.

Brain Cho is involved in synaptogenesis, the turnover, maintenance, stabilization, and restoration of synapses [63]. The functionality of synapses requires specific lipid domains in neuronal and axon membranes whose composition is critical for the correct targeting of the major membrane proteins, myelin biogenesis, cellular differentiation, signal transduction, and many other functions that depend on microdomains and specific lipid rafts. The proportion of free Cho to ECho in these membrane domains is a crucial factor in their biological activities [63]. For example, the enzymes responsible for the processing of the amyloid precursor protein (APP) to A*β* (*β*- and *γ*-secretases) reside in Cho-rich lipid rafts of plasma membrane [66]. It was suggested that a higher total Cho/phospholipid ratio in cellular membranes could affect secretase activities and determine preferential APP processing pathways [66], though *in vitro* studies suggest that Cho might impair the transcription of APP and consequently decrease the availability for its conversion to *β*A. However, it appears that this effect has no significant impact on the amount of protein (that still exceeds the capacity of the secretase system to process it) and only a slight impact on the levels of the mRNA encoding APP protein [67]. To date, there are no findings elucidating the exact role of ECho and how the ratio of free to esterified forms is able to modify secretase activity and other process associated with the amyloidogenic cascade [63]. Zana et al. [45] suggest that different sources of oxidative stress, such as NCBC, could trigger the amyloidogenic pathway, which may explain the higher A*β* 1-42/1-40 ratio we observed in the brain cortex of Cu-treated rats and in the group receiving both treatments (CuCho). 

The question as to how Cho and Cu interact to lower the production of *β*A and enhance oxidative stress and inflammation is difficult to address. Experimental evidence obtained in culture cells demonstrates that exposure of macrophages to CuSO_4_, at a level equivalent to NCBC in humans, induces SREBP-2 and consequently the expression of cholesterogenic enzymes [68], thus tentatively providing a new explanation for the apparently additive effect we observed between Cu and Cho. However, we were unable to find similar evidence in neuron or astrocyte cultures, or even in experiments conducted on live animals to explore these findings. There is yet another possibility: that the association of the two supplements (Cu and Cho) may affect the clearance of *β*A and facilitate its accumulation. Working with rabbits fed on a diet with excess Cho and inorganic Cu in their drinking water, Sparks et al. [69] proposed that Cho caused alterations in the BBB associated with an inflammatory condition and a concomitant overproduction of *β*A in the brain. In this model, Cu decreased the clearance of *β*A to the blood via inhibition of LRP at the vascular interface [69]. Impaired Cho metabolism, oxidative stress, and inflammation were all factors associated with the decreased clearance of the *β*A peptide [63, 69]. In addition, Lu et al. [14] also demonstrated that Cu exacerbates *β*A amyloid-induced neurotoxicity through a TNF-mediated inflammatory pathway. 

Though the exact mechanism(s) underlying all these effects is still unknown, it is nevertheless widely accepted that the AD pathological cascade is likely to be a 2-stage event where deposition of *β*A and neuronal pathology (tauopathy, neuronal injury, and programmed cell death, or subsequent neurodegeneration with synapse and cognitive loss) are sequential rather than simultaneous processes [70, 71]. Our model likely represents a very early step in these successive events since the screening of the damages observed in visuospatial memory revealed only slight modifications. The Barnes maze test analysis demonstrated that all groups display almost normal locomotor and exploratory activities and spatial memory retention. The behavioral modifications indicate that the animals of the control and Cho groups were fully able to acquire the necessary knowledge for the spatial task through training, whereas the Cu and CuCho groups were only partially able to do so or presented slight learning difficulties. Interestingly, these learning difficulties were more evident during the evaluation trials of Cho animals, which explored holes very distant from the escape-hole region. Nevertheless, Cho and CuCho animals both showed a minor degree (not statistically significant) of deficit in learning and spatial memory capabilities. 

Plasma levels of *β*A, particularly the *β*A 1-40/*β*A 1-42 ratio, are well-recognized biomarkers of sporadic AD [72, 73] and even indicators of early stages of the pathology [74]. However, apart from its role as a biomarker, accumulation of *β*A peptide in brain is a complex phenomenon with multiple and consecutive (sometimes unexpected) consequences [75]. In agreement with this, Tamagno et al. [76] demonstrated that oxidative stress induced by A*β*25–35 resulted in an early, significant, and time-dependent generation of free HNE (hydroxyl-nonenal) and H_2_O_2_. Also, other authors reported increased levels of oxidative stress biomarkers after A*β* exposure both *in vivo* and *in vitro* [77–79]. Our results are therefore in agreement with those of other groups reporting that oxidative stress and A*β* are linked to one another. Apparently, A*β* can induce oxidative stress [80, 81], and pro-oxidants such as NCBC can increase A*β* production [82–84] in the manner of an autostimulated process. 

The increased levels of the two main proinflammatory prostaglandins (E2 and F2*α*) are consistent with the inflammatory condition characteristic of AD [85]. This pro-inflammatory and prooxidative environment triggers the activation of calpains, whereas caspase-3 activity was not significantly stimulated under our experimental conditions. In previous papers, we also found such dissociation between the effects of inorganic Cu overload on the relative activities of the two protease systems, which—at least *in vitro* experiments—appear to depend on the extent and intensity of exposure to Cu overload [86]. However, in view of previous experimental evidence, we cannot conclude that neuronal death is actually occurring. So far, Saito et al. [87] have reported that the activation of *μ*-calpains in AD brain is not necessarily a consequence of the endstage of neuronal degeneration and may reflect a more widespread metabolic alteration that precedes and contributes to neuronal death. In fact, they observed increased activity of *μ*-calpains in the cerebellum of AD without any increase in the rate of death neurons [87]. In line with this, Nixon [88] established that different factors could lead to calpain activation triggering neurodegeneration in the early stages of AD development. Moreover, Trinchese et al. [89] also reported that calpains have many substrates that could be affected in AD patients but do not necessarily lead to immediate cell death. They also stated that *μ*-calpains are present predominantly in synapses, which is in agreement both with the fact that Cu concentration is particularly high (micromolar) in the synaptic cleft [90] and with the well-established synaptic pathology in AD [90, 91]. Nixon [88] suggested that increased activity of calpains during normal aging may also promote the development of neurological disorders and impaired calcium homeostasis, both of which could impact on the role of this cation in the function of cellular membrane receptors and metallosignaling in brain [91].

Finally, in discussing the validity and/or limitations of our experimental system, it is necessary to consider the level of the supplementation with Cu using oral administration. Our experimental conditions were based on previous work [13, 14] and resemble the Cu levels commonly found as a consequence of involuntary exposure through air, food and water pollution [16, 92–94], ingestion of dietary mineral supplements, exposure of professionals engaged in agrochemical activities [6, 11, 95], neurodegenerative patients and their first-degree relatives, or female users of Cu-based intrauterine devices [17, 19]. Studies performed in rats demonstrated that Cu metabolism and homeostasis are essentially identical to those in humans [96]. In terms of dosage, the rats from the groups supplemented with Cu received 1.06 ± 0.11 mg Cu/Kg/day (including Cu acquire from food and water ingestion). From the available data in humans, we can assume that general population are receiving 0.16 to 0.98 EAR or RDA which is equivalent to 0.3 to 1.5 mg Cu/kg body weight [97]. Very probably, humans are exposed to several types of Cu-based compounds of different chemical structures with differences in their physical stabilities, solubility, absorption capacities, life's times into the organism, and many other particularities related to their excretion or bioaccumulation rates. However, only inorganic Cu should be dangerous for its probable role as causative factor for neurodegeneration [3, 6, 8–10]. Thus, we think that there are a lot of questions to be answered before drawing a realistic conclusion about the comparisons between our experimental conditions and the actual human expose to Cu.

## 5. Conclusions

 In conclusion, this *in vivo* study reveals that the association of inorganic Cu and Cho gives rise to a prooxidative and proinflammatory environment more pronounced than that produced by Cu and Cho administered alone. As described before, the combination of these two factors is common in many human populations. We suggest that the biochemical changes observed, in particular, oxidative stress, inflammation, and the higher A*β* 1-42/1-40 ratio in the cortex of rats fed on Cu + Cho (CuCho), could constitute the initial stages of the development of neurodegenerative disease. In view of the abundant evidence of disturbed Cu homeostasis in AD [7, 56, 98, 99], we strongly recommend more in-depth studies on the mechanism(s) responsible for the pro-neurodegenerative effect(s) of the association between NCBC and Cho. Furthermore, it is recommended that the present experimental evidence be used to promote the investigation of the emerging biomarkers—such as those examined in this work—to be applied in peripheral plasma as predictive tool(s) in high-risk populations. 

## Figures and Tables

**Table 1:** Copper (Cu) levels in animals fed with the experimental diets.

Diets	Cu (ng/mg prot)					
	Plasma		Brain			
	Total	NCBC	Cortex		Hippocampus	
			Total	NCBC	Total	NCBC
Control	18.5 ± 0.8^a^	0.95 ± 0.11^a^	5.5 ± 0.2^a^	0.25 ± 0.04^a^	4.8 ± 0.1^a^	0.27 ± 0.04^a^
Cu	23.2 ± 0.7^b^	1.76 ± 0.15^b^	7.1 ± 0.1^b^	0.27 ± 0.02^a^	6.1 ± 0.1^b^	0.33 ± 0.02^b^
Cho	18.1 ± 1.0^a^	0.91 ± 0.05^a^	5.4 ± 0.2^a^	0.24 ± 0.04^a^	5.1 ± 0.2^a^	0.25 ± 0.04^a^
CuCho	22.9 ± 0.6^b^	1.78 ± 0.10^b^	7.0 ± 0.1^b^	0.29 ± 0.03^a^	6.2 ± 0.04^b^	0.35 ± 0.03^b^

Cu content was determined after mineral digestion of the samples by atomic absorption spectrometry as described in Section 2.5. Results are the mean of 10 independent measurements analyzed in triplicate ± SD. Comparisons between data were performed by ANOVA + Tukey test at *P* < 0.01. Statistical differences among the experimental diets were indicated with distinct superscript letters (values within the same column with different superscript letters are statistically significant between them).

**Table 2:** Esterfied (ECho) and free (FCho) cholesterol levels in animals fed with the experimental diets.

Diets	Cho (nmoles/mg·prot)								
		Plasma		Brain zones					
				Cortex			Hippocampus		
	Esterified	Free	FCho/Echo	Esterified	Free	FCho/Echo	Esterified	Free	FCho/Echo
C	16.4 ± 0.3^a^	10.6 ± 0.2^a^	0.64 ± 0.05^a^	28.2 ± 1.9^a^	257.8 ± 10.6^a^	9.14 ± 0.71^a^	28.7 ± 2.2^a^	247.1 ± 9.7^a^	8.61 ± 0.60^a^
Cu	16.8 ± 0.4^a^	11.7 ± 0.3^a^	0.70 ± 0.05^a^	35.8 ± 2.2^b^	260.2 ± 7.3^a^	7.26 ± 0.42^b^	39.2 ± 1.8^b^	252.1 ± 11.4^a^	6.43 ± 0.33^b^
Cho	22.2 ± 0.4^b^	11.5 ± 0.4^a^	0.51 ± 0.03^c^	30.6 ± 2.1^a^	281.3 ± 12.2^b^	9.19 ± 0.55^a^	28.0 ± 1.7^a^	269.4 ± 11.4^b^	9.62 ± 0.21^c^
CuCho	18.3 ± 0.5^a^	15.5 ± 0.5^c^	0.85 ± 0.05^d^	70.0 ± 3.1^c^	225.3 ± 15.0^c^	3.21 ± 0.13^c^	77.5 ± 2.7^c^	233.6 ± 11.5^a^	3.01 ± 0.11^d^

Cho and Cho-esters contents were determined enzymatically after HP-TLC as described in Section 2.7. Results are the mean of 10 independent measurements analyzed in triplicate ± SD. Comparisons between data were performed by ANOVA + Tukey test at *P* < 0.01. Statistical differences among the experimental diets were indicated with distinct superscript letters (values within the same column with different superscript letters are statistically significant between them).

**Table 3:** Total glutathione content, ratio oxidized (GSSG)/reduced (GSH) glutathione, glutathione reductase activity (GR), and concentration of *α*-tocopherol (*α*-Toc) in plasma and brain cortex and hippocampus from the different experimental groups.

Diets		Plasma		Brain zones							
				Cortex				Hippocampus			
	GSH + GSSG	GSSG/GSH	*α*-Toc	GSH + GSSG	GSSG/GSH	GR	*α*-Toc	GSH + GSSG	GSSG/GSH	GR	*α*-Toc
C	13.0 ± 0.05^a^	0.06 ± 0.01^a^	20.9 ± 0.06^a^	796.5 ± 33.3^a^	0.11 ± 0.01^a^	184.6 ± 7.2^a^	0.35 ± 0.02^a^	701.0 ± 30.5^a^	0.08 ± 0.01^a^	236.7 ± 5.6^a^	0.27 ± 0.01^a^
Cu	13.5 ± 0.11^a^	0.08 ± 0.02^a^	18.8 ± 0.12^a^	808.8 ± 41.0^a^	0.12 ± 0.02^a^	200.4 ± 7.9^b^	0.36 ± 0.02^a^	715.8 ± 41.1^a^	0.10 ± 0.02^a^	250.2 ± 4.6^b^	0.25 ± 0.02^a^
Cho	12.7 ± 0.20^a^	0.06 ± 0.02^a^	19.5 ± 0.10^a^	835.2 ± 41.2^a^	0.10 ± 0.02^a^	185.67 ± 7.8^a^	0.35 ± 0.03^a^	728.4 ± 38.5^a^	0.07 ± 0.01^a^	240.8 ± 3.2^a^	0.26 ± 0.03^a^
CuCho	15.8 ± 0.14^b^	0.12 ± 0.03^b^	15.2 ± 0.08^b^	889.2 ± 41.3^b^	0.16 ± 0.01^b^	222.33 ± 6.5^c^	0.29 ± 0.01^b^	735.8 ± 31.7^a^	0.19 ± 0.01^b^	284.5 ± 10.3^c^	0.18 ± 0.02^b^

Results (mean of 10 animals assayed in triplicate ± SD) were obtained as described in Sections 2.9.1, 2.9.2, and 2.9.3. Total glutathione (GSH + GSSG) and GSH are expressed as *μ*moles/mg protein. GR activity was expressed as U/mg·prot·min. *α*-Toc was calculated in nmoles/mg·protein. Results statistically significantly different (*P* < 0.01) are indicated with distinct superscript letters (values within the same column with different superscript letters are statistically significant between them).

**Table 4:** A*β* (1–40 and 1–42) peptide concentrations and the ratio A*β* (1–42)/(1–40) in plasma and in brain regions (cortex and hippocampus) in rats fed with the experimental diets.

Treatments		Plasma		Brain zones					
				Cortex			Hippocampus		
	Aβ (1–40)	Aβ (1–42)	Aβ (1–42)/(1–40)·10^2^	Aβ (1–40)	Aβ (1–42)	Aβ (1–42)/(1–40)·10^2^	Aβ (1–40)	Aβ (1–42)	Aβ (1–42)/(1–40)·10^2^
C	82.65 ± 1.91^a^	7.06 ± 0.15^a^	8.55 ± 0.15^a^	59.49 ± 1.91^a^	8.15 ± 0.33^a^	7.30 ± 0.16^a^	31.56 ± 1.85^a^	5.01 ± 0.26^a^	6.30 ± 0.12^a^
Cu	94.62 ± 1.55^b^	8.44 ± 0.31^b^	8.92 ± 0.23^a^	88.87 ± 1.44^b^	9.66 ± 0.45^a^	9.20 ± 0.22^b^	41.18 ± 1.81^b^	6.24 ± 0.20^a^	6.60 ± 0.24^a^
Cho	112.05 ± 2.33^c^	9.95 ± 0.33^c^	8.88 ± 0.33^a^	115.58 ± 1.29^c^	12.04 ± 0.28^b^	9.60 ± 0.18^b^	72.65 ± 2.22^c^	7.49 ± 0.18^b^	9.70 ± 0.15^b^
CuCho	132.76 ± 2.40^d^	12.56 ± 0.22^d^	9.46 ± 0.28^a^	176.40 ± 2.03^d^	15.74 ± 0.25^c^	11.20 ± 0.26^c^	87.76 ± 1.44^d^	8.82 ± 0.21^c^	9.97 ± 0.22^b^

Results (mean of 10 animals assayed in triplicate ± SD) were obtained as described in Section 2.12.1. Individual peptides are expressed as picomoles/L (plasma) or picomoles/mg protein (brain homogenates). Results statistically significantly different (*P* < 0.01) are indicated with distinct superscript letters (values within the same column with different superscript letters are statistically significant between them).
